# Trends of early infant feedings practices after counseling in infant born to HIV positive women in Yaoundé, Cameroon

**DOI:** 10.11604/pamj.2014.17.282.3500

**Published:** 2014-04-15

**Authors:** Anne Esther Njom Nlend, Bernadette Bagfegue Ekani

**Affiliations:** 1 National Social Insurance Fund Hospital, Department of Pediatrics; 2 Association Camerounaise d'Aide aux Personnes et Familles Affectées par le VIH/SIDA

**Keywords:** HIV, infant feeding, counseling

## Abstract

The objective is to describe the trends of infant feedings choices in HIV context after infant feeding counseling. Descriptive retrospective study: Infant feeding counseling (IFC) sessions were offered to HIV pregnant women by the same team of counselors from April 2008 to December 2012. Counseling content was promoting either exclusive breastfeeding (EBF) or exclusive formula feeding (EFF) prior to 2010. Later on, versus EBF+ antiretroviral (ARV) drug given either to the mother or the infant or EFF was the gold standard. Mixed feeding was prohibited. Infants feeding were practices recorded at the first post natal visit. Main measurement: rate of EBF/ EFF per year and period. We included a total of 1114 live-born babies. During the five year the overall rate of EBF and EFF stood at 41% and 59% respectively. The rate of EBF/EFF was recorded as follow: varies from 25/75% in year one to 52/48% in year five(p≤0.001). The rate of mixed was virtually cancelled during the same period, 3/237 (1.2%) in year one to period 1/165 (0.6%) in the latest period. In conclusion, in Yaoundé, there is a slight increase in breastfeeding rate among HIV exposed infants during the first two months of life. Further investigations are required to confirm this tendency and analyze the new features of breastfeeding practices.

## Introduction

The issue of infant feeding remains of up most importance for child survival [1]. In the HIV context, its practices have been controversial. For long, the rate of replacement feeding has been quite high in many African urban areas [2–4] due to misconceptions and or poor trained counselors. Recently, critical changes have occurred in preventing HIV-1 transmission through breastmilk, making it safer under coverage of antiretroviral therapy (ART). The evidence and benefits of antiretroviral drugs (ARV) taken either by the mother or the baby [5] to prevent HIV transmission through breastfeeding have resulted in modifying the World Health Organization(WHO) infant feeding guidelines in HIV context [6]. Consequently, the content of infant feeding counseling (IFC) has changed making breastfeeding coupled to ARV mandated for any HIV nursing mother, whereas breastfeeding without ARV is no longer ethical, at all. From our observation, in a single referral site for care of HIV people, these changes have created a new paradigm for HIV pregnant women, whose trends to opt and practice breastfeeding seem to increase. Upon this hypothesis, we decided to analyze the features of feeding mode of HIV exposed infant, from 2008 to 2012, with the objective to determine whether current WHO infant feeding guidelines, adopted in the country have leveraged the rate of breastfeeding.

## Methods

**Ethical clearance:** This study received the approval of the national committee on research on human health in Cameroon. Verbal consent was given by the mothers. Confidentiality and anonymity of all processes were ensured for all respondents.

**Study Design:** We conducted a retrospective analysis.

**Study site:**The Centre Hospitalier d'ESSOS, an approved center for ART treatment was hosting the study.

**Study population:** Mothers and baby pairs followed up between 2008 and 2012 were included in this study. We include mothers who had benefit of counseling sessions for choices during pregnancy and infants seen during the first two months of life with a recorded feeding option since birth.

**Procedures:** During pregnancy, infant feeding counseling (IFC) sessions to mothers were offered following the national guidelines. Initially, the counseling content was presenting two main options to the eligible mothers which were either exclusive breastfeeding or exclusive formula feeding, mixed feeding was prohibited. After 2010, following the revisions of WHO, IFC guidelines, we modified our counseling content and trained the counselors consequently. The new counseling content was highlighting systematic uptake of ARV by the lactating mothers or infants in order to lessen the risks of acquired HIV through breastfeeding. This new concept resulted in alleviating the arguments against breastfeeding for those mothers, whereas the messages about formula feeding and mixed feeding remained unchanged. During all the project, tins of breastmilk substitutes could be provided under specific restricted conditions with respect to the marketing code of breastmilk substitutes in order to avoid any kind of spill over.

**Definition of terms:** EBF was defined as intake of breastmilk only except vitamins. Mixed feeding was defined by uptake of both breastmilk and formula. Formula feeding was defined by feeding using infant commercial formula or home prepared formula.

After birth, HIV exposed infants were registered and seen at the first follow up visit (not later than 2 months of life) with a recorded option practiced since birth.

### Data management

**Data sources and collection:** Options practices since birth upon mothers’ declaration were stored in a excel sheet data base.

**Variables and data analysis:** This short report focuses on the evolutive rate of breastfeeding and FF from 2008 to 2012. HIV-1 exposed infants with notified infant feeding choices were included in the analysis. Assignment to an infant feeding group (formula-fed, breastfed, mixed fed) was based on the feeding option practiced since birth as reported by the mothers during the first post natal visit. Rate of breastfeeding/formula feeding, expressed in percentages, were compared using the chi-square test. Comparisons yielding a p-value of 0.05 were considered as statistically significant.

## Results

**Study population:** We included a total of 1114 live-born babies. Of whom 237 were registered in 2008, 299 in 2009, 279 in 2010, 147 in 2011 and 152 in 2012. Mother's characteristics were similar along the time. The median age of the women was 27 years, the majority (63%) had undergone secondary education, 63% were cohabiting with a partner, about 1 in 10 had attained tertiary education and almost 75% of women had disclosed their status to their partner. The household conditions of our respondents was presenting the following features: piped water was available for most part of them (89%), the majority were using gas or paraffin as combustible energy for cooking (>75%), and nearly the third were using modern latrines.

**Figure 1:**
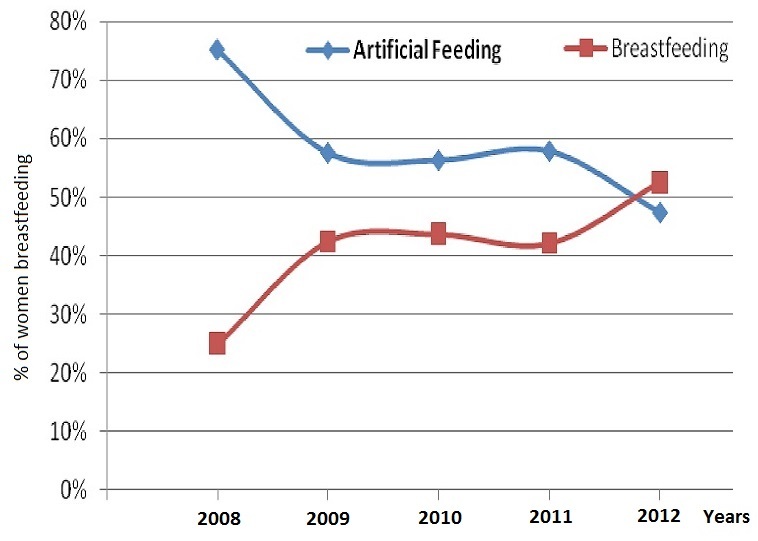
Trends of infant feeding at 2 months, in infant born to HIV positive mothers in Yaoundé in one single site from year 2008 to year 2012

**Trends of Infant feedings:** The rate of breastfeeding varies from 25% in year one to 52% in year five (Figure 1) (p <0.001). The rate of mixed was virtually cancelled during the same period moving from 3/237 (1.2%) in year one to period 1/165 (0.6%) in the latest period.

## Discussion

The main result in this study is an increase trend to BF along the time concomitantly with the reduction of formula feeding among our women, as well as the virtual elimination of mixed feeding. At the end of the period, less than ½ women are still opting for EFF, contrary to the beginning and to data posted from similar urban places [4, 7]. The most laudable fact is the cancelling of mixed feeding far difficult to achieve in many settings [8]. However, this feature started earlier before the formal change of our counseling content and has been slightly maintain year after year. We are assuming that this tendency may be put on the improvement of quality counseling as well as on the change of counseling content, as IFC is known to be a key intervention for those mothers [9]. Our main assumption is that the new guidelines are certainty making the BF choices safer for the mother, allowing them to normally breastfed their child and to avoid any kind of stigmatization [10]. This may surely need further investigation notably qualitative and deep interviews towards both mothers and counselors, as we were amazed to find many women switching from formula feeding to breastfeeding at their second pregnancy in the HIV conditions.

## Conclusion

There is a slow changing trend in early infant feeding practices in infant born to HIV positive women in Yaoundé. Further studies are required to deeply analyze these new features.
